# Mediastinal abscess after endobronchial ultrasound with transbronchial needle aspiration: a case report

**DOI:** 10.1186/1749-8090-5-33

**Published:** 2010-05-05

**Authors:** Susan D Moffatt-Bruce, Patrick Ross

**Affiliations:** 1 Division of Cardiothoracic Surgery, Department of Surgery, The Ohio State University, Columbus, Ohio, USA, 43210

## Abstract

Endobronchial ultrasound (EBUS) with transbronchial needle aspiration is now becoming widely accepted as a preferred staging technique. It has been perceived as a non-invasive and well tolerated procedure with minimal complications. We report the development and treatment of a severe complication that developed 2 weeks after the initial procedure in the form of a complex mediastinal abscess. EBUS although useful in its non-invasive application for diagnosing mediastinal or hilar disease, must be regarded with caution since the potential exists to develop severe complications.

## Case Report

**Figure 1:**
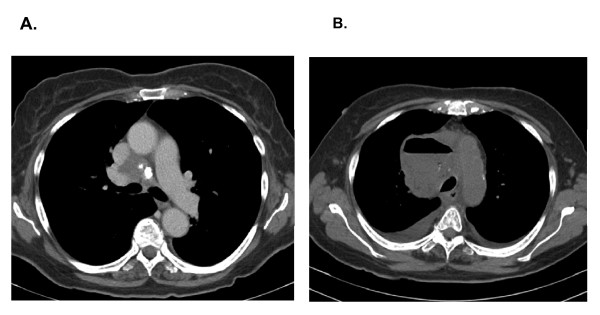
**Computed Tomography Scan of Mediastinal Pathology**. **A**. The patient had a preoperative CT scan that revealed mediastinal adenopathy. **B**. Two weeks after EBUS and transbronchial needle aspiration, the patient presented with a complex mediastinal abscess.

An 89 year old woman presented to thoracic surgery clinic for the evaluation of mediastinal adenopathy. She had a history of a frontal meningioma that had been treated with radiotherapy 9 years earlier and also had an undiagnosed renal lesion that was being followed with sequential imaging. In the course of this follow up, a computed tomography (CT) scan of the chest had been obtained which revealed mediastinal adenopathy (Figure 1A). Although not hypermetabolic on positive emission tomography (PET), the indeterminate etiology necessitated endobronchial ultrasound (EBUS) with transbronchial aspiration.

The patient underwent an uneventful auto fluorescent bronchoscopy and EBUS (Evis Exera Olympus BF-UC160F-OL8). Appropriate ultrasound lymph node criteria were met in the subcarinal area and four transbronchial aspirates were taken with a 22-gauge aspirating needle with syringe model NA-201SX-4022-A (Olympus, Center Valley, PA). The pathology revealed lymphocytes and benign elements of respiratory mucosa. No malignancy was identified. Approximately 10 days after the biopsy, the patient reported a fever. The patient declined admission and was placed on oral antibiotics. Fourteen days after the procedure the patient presented to the local emergency department complaining of shortness of breath and fever. A CT scan of the chest revealed an air-fluid filled 7.5 × 7.6 cm right paratracheal mass that was displacing the aorta (Figure 1B.) She was started on empiric intravenous antibiotics and transferred to our center. The patient was stable upon admission but she subsequently developed atrial fibrillation and hypotension. The patient was taken to the operating room for bronchoscopy, esophagoscopy, right video assisted thoracoscopy and drainage of a mediastinal abscess. Broad spectrum antibiotics were administered until the cultures from the abscess documented alpha stretptococcus and Diphtheroids. The final pathology of the drained abscess revealed an organizing abscess cavity and granulation tissue with no malignancy detected. Post-operatively, the patient developed a sepsis syndrome involving both respiratory and renal failure requiring prolonged ventilation and dialysis. The patient was weaned from the ventilator on day 10 post operatively. She was discharged home on renal replacement therapy. At her four month follow up her mediastinal abscess had completely resolved on repeat CT scan; she had fully recovered from her renal failure.

## Discussion

Obtaining a tissue diagnosis of mediastinal adenopathy can be challenging. Transbronchial needle aspiration (TBNA) that has been performed "blindly" has been in existence for more than 20 years but has been little used likely due to its static nature and low sensitivity [1,2]. Until recently, mediastinal adenopathy has required surgical intervention in the form of a mediastinoscopy or video-assisted thoracoscopic mediastinal lymph node dissection. With the introduction of a radial probe, which uses a radial scanning ultrasonic miniprobe (EBUS) inserted through the bronchoscope, sensitivity for diagnosing mediastinal nodes appeared to have increased. Whilst this devise was good for discerning vascular structures, it had to be withdrawn at the time of the actual biopsy. The development of the convex probe (CP-EBUS, XBF-UC160F-OL8/BC-UC260F-OL8; Olympus Medical Systems, Center Valley, PA) however has overcome this problem with the ability to simultaneously use the ultrasound probe to visualize sampling the node. Recently reviewed literature speaks to a sensitivity range of 85-100% and a negative predictive value of 11-97% in diagnosing mediastinal adenopathy associated with lung cancer and similar success in diagnosing sarcoidosis and lymphoma [3]. Authors have also reported success in terms of cancelling transthoracic needle biopsies and surgical diagnostic procedures in up to 47% of cases [4]. it is therefore reasonable to suggest that for the evaluation of mediastinal adenopathy, EBUS directed biopsy, is fast becoming the preferred method of diagnosis [5,6].

Part of the attraction of EBUS and transbronchial biopsy has been the lack of reported complications. Presumably, the EBUS component is thought to have eliminated or reduce the potential of complications that were rarely associated with "blind" or conventional TBNA such as aortic puncture, pneumomediastinum and chylothorax [7,8]. Large centers have described their learning curve experience and report that after 10 procedures, the sensitivity of EBUS with transbronchial biopsy can be as high as 96% with an accuracy of 97% [6]. Despite the learning curve however, no complications were reported in over 100 procedures [6]. Similarly, a review of the literature pertaining to a recent 24 month period (2007-2008), no surgical complications were reported, with the exception of sedation related issues [3,6]. A very recent case report from a large academic center presents two infectious complications from endobronchial ultrasound transbronchial needle aspiration [7]. The first was a pericardial effusion that was positive for *Actinomyces odontolyticus *and *Streptococcus mutans*. Full recovery followed percutaneous drainage and antibiotic therapy. The second was a lung abscess that was treated with prolonged antibiotics. Neither complication required surgical intervention or a prolonged hospitalization.

The case presented herein highlights the potential for a serious complication with innovative technology. Upon review of the patient's transbronchial aspirates, the pathology was non-diagnostic and had not been sent for culture. As a result of this case, it is now our practice to send all EBUS aspirates for culture in addition to obtaining bronchial aspirates. As a thoracic surgery group, we have now completed over 80 EBUS and transbronchial aspirates without additional complications. As a result of this complicated case, however, our clinical awareness has been heightened. We would encourage caution with a technique often associated with few complications.

## Consent

Written informed consent was obtained from the patient for publication of this case report and any accompanying images. A copy of the written consent is available for review by the Editor-in-Chief of this journal.

## Abbreviations

(EBUS): Endobronchial ultrasound; (CT): Computed tomography; (PET): Positive Emission Tomography; (TBNA): Transbronchial needle aspiration.

## Competing interests

Dr. Moffatt-Bruce and Dr. Ross declare that they do not have any financial or non-financial competing interests relative to this case report.

## Authors' contributions

PR was the primary caregiver for this patient and reviewed the manuscript. SMB also cared for this patient and drafted and completed the manuscript. Both authors read and approved the final manuscript.
